# Repetitive sex change in the stony coral *Herpolitha limax* across a wide geographic range

**DOI:** 10.1038/s41598-018-37619-y

**Published:** 2019-02-27

**Authors:** Lee Eyal-Shaham, Gal Eyal, Kazuhiko Sakai, Yoko Nozawa, Saki Harii, Frederic Sinniger, Omri Bronstein, Or Ben-Zvi, Tom Shlesinger, Yossi Loya

**Affiliations:** 1 Tel Aviv University, School of Zoology, Tel Aviv, 6997801 Israel; 2 The Interuniversity Institute for Marine Sciences, P.O. Box 469, Eilat, 8810369 Israel; 3 Tropical Biosphere Research Center, University of the Ryukyus 3422 Sesoko, Motobu, Okinawa 905-0227 Japan; 4 Biodiversity Research Center, Academia Sinica, Taipei, Taiwan

## Abstract

Sex change has been widely studied in animals and plants. However, the conditions favoring sex change, its mode and timing remain poorly known. Here, for the first time in stony corals, we report on a protandrous (youngest individuals are males) repetitive sex change exhibited by the fungiid coral *Herpolitha limax* across large spatial scales (the coral reefs of Japan, Jordan and Israel) and temporal scales (2004–2017). In contrast to most corals, this species is a daytime spawner (08:00–10:00 AM) that spawned at the same time/same date across all the study sites. The sporadically scattered populations of *H. limax* among the coral reefs of Eilat (Israel) and Aqaba (Jordan) exhibited significantly slower growth, earlier sex change, and lower percentages of reproduction and sex change in comparison to the densely aggregated populations in Okinawa (Japan). At all sites, sex ratio varied among years, but was almost always biased towards maleness. Growth rate decreased with size. We conclude that comparable to dioecious plants that display labile sexuality in response to energetic and/or environmental constraints, the repetitive sex change displayed by *H. limax* increases its overall fitness reinforcing the important role of reproductive plasticity in the Phylum Cnidaria in determining their evolutionary success.

## Introduction

Sexual reproduction is a fundamental aspect of life that manifests in the diverse sexual systems occurring in nature. Despite its high energetic expenditure, the evolutionary advantages of sexual reproduction outweigh its cost by increasing progeny fitness and genetic diversity^1^. However, because resources are finite, the possible costs of reproduction may reduce the survival probability or growth rate of the individual. As such, the rules governing reproductive allocation are viewed in a benefit–cost framework^2^. This fundamental trade-off has led individuals to adopt specific tactics to attain reproduction. According to sex-allocation theory^3^, natural selection favors parents that modify their investment in male and female functions in such a way that it maximizes the parent’s fitness^3,4^. Among the broad variety of sexual systems, sequential hermaphroditism (sex change) represents the most extreme form of sex-allocation, through altering sexual function expression (reviewed by^5^). In most cases of sequential hermaphroditism, sex change appears to occur only once in a lifetime^3,6^, either from female to male (protogyny) or from male to female (protandry). However, a minority of species have proven to have the ability to change sex bi-directionally (i.e. the occurrence of sex change from female to male and from male to female in the same population), or even repetitively (reviewed by^7^). This phenomenon described both in animals^6–15^ and plants^6,16–19^ presumably conveys a selective advantage to an individual by increasing its reproductive potential.

Stony corals (scleractinians), the frame-builders of coral reefs, are sessile organisms that possess ecological features typical of both plants and animals^13^. The sexual reproduction in scleractinian corals involves two main sexual systems: colonies are either predominately out-crossing, simultaneous hermaphrodites, with each polyp having both male and female functions; or polyps within a colony express only one sex throughout their life and such colonies are thus either male or female and the species are gonochoric (dioecious) (reviewed by^20^). Nonetheless, atypical sexual systems of mixed or contrasting patterns of sexuality have been reported from various coral species^21,22^ including that of corals exhibiting sex change. Protandrous sequential hermaphroditism was documented in four solitary fungiid species (*Ctenactis echinata, C. crassa, Fungia repanda* and *F. scruposa*)^13^, two of which (*C. echinata* and *C. crassa*) changed sex bi-directionally^13,23^.

The most influential model of sex change is the size-advantage hypothesis model (SAH), which predicts sex change when the reproductive success of one sex increases more rapidly with size than the reproductive success of the opposite sex^3,7,10,24–26^. The direction of sex change (protandrous or protogynous) is determined by the relative reproductive success over the course of a lifetime for the two sexes. If male fitness increases with size (age) at a higher rate than female fitness, sex change will be protogynous. The reverse holds true for protandrous sex change^5,13,24,25,27,28^. However, large size does not always confer a greater reproductive advantage upon one sex than the other^29^ and, therefore, additional explanations are required for the existence of sex change. Alternative models, developed primarily as models based on environmental sex determination in plants^16^, have suggested that sex change can be triggered by epigenetic cues (environmental or social information) (reviewed by^5,7^). Freeman *et al*.^16^, and Korpelainen^18^ have demonstrated that environmental factors are strongly correlated with the sexual expression of the individual. For example, harsh environmental conditions were demonstrated to induce a shift from female to male function in the shrub *Atriplex canescens* as an adaptive strategy to avoid the high cost of female reproduction^16,30^. Similarly, age, injury, and disease have been shown to alter sexual expression towards maleness^13,16^. The magnitude and direction of sex change may be influenced by population demography^31^. Evidence of this is more common in mobile animals that exhibit social control, and the phenomenon has been widely explored in fish, the largest vertebrate group in which this phenomenon occurs^7,32,33^. However, in sessile or very low-mobility animals that lack any obvious social interactions, such as scleractinian corals, the cues regulating sex change may be different.

The similarity in some life-functions and life-history traits between scleractinian corals and certain plants^13^, enabled us to study the labile nature of sex expression from a different perspective and to examine a variety of theoretical and empirical studies of sex change on these sessile organisms. In this wide-scale study, we examined the reproductive behavior of individual corals representing a broad array of size groups over an extended period of time (13 years), in three distant geographical areas (the coral reefs of Eilat, Israel, Aqaba, Jordan and Okinawa, Japan), exhibiting divergent environmental conditions. The detailed data obtained in this study during 2004–2017 allowed us to examine both the long-term reproductive patterns of the fungiid coral *Herpolitha limax*, and the labile sexual expression it demonstrates in light of the local prevailing environmental conditions.

## Results

### Reproduction of *Herpolitha limax*

*H. limax* belongs to the family Fungiidae, which consists of solitary, largely single-polyped, “free-living” species (i.e. not attached to the substrate). Out of the 28 fungiid species whose sexuality were thought to be of the gonochoric type^20^, four were found to change sex, and among these, two species change sex bi-directionally^13,23^. In the Gulf of Eilat/Aqaba (GoE/A), in both the Eilat and Aqaba reefs, the species appear in a dispersed manner and occur mostly at a depth range of 5–50 m. The abundance of *H. limax*, however, is higher in Aqaba than in Eilat (L.E-S., YL, pers. obs). In contrast, in the coral reefs of Okinawa, the *H. limax* population is mainly concentrated at a few sites inhabited by thousands of individuals, which are densely distributed between 5–20 m depth.

Observations on *H. limax* began in July 2004, in Okinawa, Japan and a few years later in Eilat and Aqaba, within the framework of a wide-scale study of reproduction patterns in the Fungiidae family. However, detailed data on the mode and timing of reproduction of *H. limax* populations, based on large sample sizes, commenced only in 2012 in Eilat and 2013 and 2014 in Aqaba and Okinawa, respectively (see Supplementary Material for further details).

**Figure 1:**
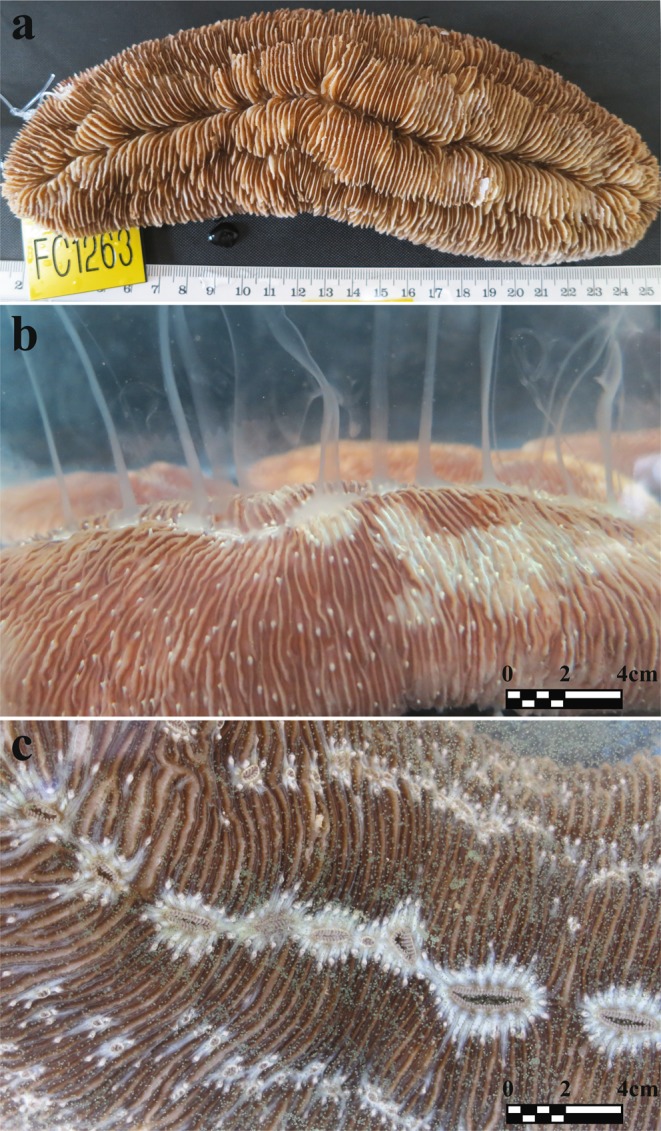
*Herpolitha limax*: a sequential hermaphrodite daytime spawner. (**a**) An example of a tagged individual (using a numeric plastic tag attached by a nylon fishing cord inserted through a thin hole drilled at the edge of the coral skeleton) monitored throughout the span of the study. A total of 480 individuals were monitored at the three study sites (175, 93 and 212 specimens in Okinawa, Eilat and Aqaba, respectively). (**b**) Male shedding spurts of sperm through multiple mouth openings. (**c**) Female shedding eggs through multiple mouth openings. In contrast to most corals, this species is a daytime spawner (releasing gametes between 8 am to 10 am for 5–7 consecutive days, five days after the full moon, of June-August or July-September). In all three study sites *H. limax* released gametes during the same months, same lunar age and at exactly the same time, in consecutive years.

**Figure 2:**
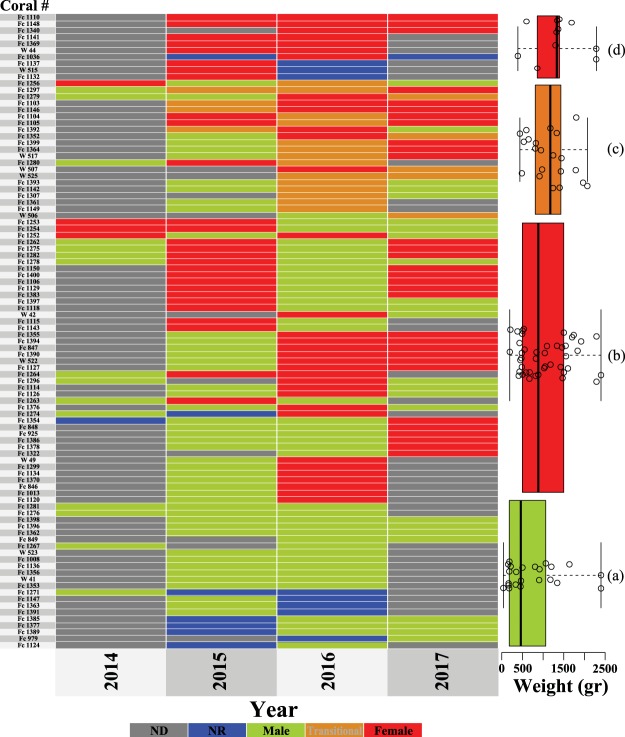
*Herpolitha limax* (Okinawa, Japan): a heat-map displaying ‘expressed sexuality’ per individual coral (i.e corals whose reproduction had been studied for two years or more) throughout the study years. The cluster-aggregated boxplots to the right of the heat-map display the individual coral weight (g). Centerlines show the medians; box limits indicate the 25^th^ and 75^th^ percentiles; whiskers extend to min and max values and the range is colored in accordance with the legend below the heat-map; data points are represented by blank circles. n (from top to bottom) = 10, 21, 43, 22 individual coral weights. The color of each cell in the heat-map corresponds to the legend below it. Gray cells (ND) correspond to missing data and blue cells (NR) to non-reproductive coral. The rows (individual corals) are ordered by patterns of sexuality. Boxplot (**a**) – represents individuals that were documented only as ‘males’ throughout the study; Boxplot (**b**) – represents individuals that changed sex at least once but were not documented as having both gametes simultaneously (i.e. transitional); Boxplot (**c**) – represents individuals that were documented as containing both gametes simultaneously (i.e. transitional) at least once during the study. Boxplot (**d**) - represents individuals that were documented only as ‘females’ throughout the study.

In contrast to other fungiids, as well as the majority of scleractinian corals, which are typically night spawners, *H. limax* is a daytime spawner (Fig. 1a) that releases gametes of separate sexes between 8 am to 10 am for 5–7 consecutive days, starting five days after the full moon, in June-August or in July-September (apparently depending on the lunar periodicity of the given year). This pattern repeated itself at all three study sites in consecutive years, with the corals spawning at precisely the same months, lunar age and time. The female gametes are small (~50 µm), viewed as tiny dots floating in the water volume, while the male gametes appear as a milky cloud, causing the water to appear murky (Fig. 1b,c). Overall, the reproduction of *H. limax* was highly synchronized at all study sites, both locally and globally (timing and date of reproduction). Most of the reproductive individuals spawned for 2–4 days within the expected reproduction period of each month. Some individuals spawned across two consecutive months, while others spawned for one month only. The vast majority of corals released gametes of only one sex each season. However, at the Aqaba and Okinawa study sites some corals were found to release both sex gametes during one breeding season. We termed this reproductive state ‘transitional’ (T) (see Table 1). Release of the different gametes was observed either at a one month interval; or, more rarely, during the same reproduction month, at a one-day interval. Throughout the study years, only one individual (out of 79 corals examined in 2017) was observed to release both gametes simultaneously. No similar ‘transitional state’ was observed in Eilat. The percentages of reproduction were higher in Okinawa than in Eilat and Aqaba (Table 1). Of the Eilat breeding population (i.e. corals for which reproductive data for more than two years were available), 19% had changed sex in either directions throughout the years (either from male to female or from female to male). In Okinawa, 66% of the breeding population had changed sex in both directions, and in Aqaba 11% of the breeding population exhibited sex change in both directions. Repetitive sex change was documented in Eilat (10%) and Okinawa (29%) but was seemingly absent in Aqaba (most probably due to the lack of repeated observations) (Fig. 2; Table 2; Supplementary Material Figs S1, S2). In winter 2014–2015, an extremely destructive flood impacted the north-eastern part of the GoE/A, causing heavy mortality to corals in Aqaba that were located on the shallow reef near the local Marine Science Station. Unfortunately, 108 individuals among the tagged corals died in the flood, whose consequences prevented us from fully examining the continuity of reproduction. The sampled population of 2015 therefore comprised 89 new individual corals and only 28 survivors that had been recorded in previous years.

**Table 1:** *Herpolitha limax*: Population reproductive traits during 2012–2017 in Okinawa, Aqaba and Eilat. Sex-ratio: M:F:T - Male: Female: Transitional state.

Site	Okinawa				Aqaba		Eilat					
Year	2014	2015	2016	2017	2013	2015	2012	2013	2014	2015	2016	2017
Sample size	n = 58	n = 89	n = 103	n = 79	n = 123	n = 117	n = 48	n = 54	n = 45	n = 66	n = 52	n = 55
Number (n) and sex of corals observed breeding	29 M	47 M	50 M	30 M	31 M	18 M	25 M	16 M	7 M	23 M	13 M	20 M
	6 F	31 F	31 F	34 F	25 F	9 F		10 F		7 F	1 F	4 F
		4 T	14 T	4 T	3 T	2 T						
No. of non-reproductive (NR) corals	23	7	8	11	64	88	23	28	38	36	38	31
Sex-ratio (M:F:T)	1:0.2	1:0.65:0.08	1:0.62:0.28	0.88:1:0.11	1:0.8:0.09	1:0.5:0.11	1:0	1:0.62	1:0	1:0.3	1:0.07	1:0.2
% reproduction	60	92	92	86	47	24	52	48	15	45	27	44
% corals releasing both gametes (T)	0	4	15	6	5	6	0	0	0	0	0	0

**Table 2:** *Herpolitha limax*: Life-history characteristics.

Variable	Okinawa	Aqaba	Eilat
Direction of sex change	Protandry, repetitive	Protandry, bidirectional	Protandry, repetitive
Total # of corals examined	175	212	93
Breeding population^*^	97	54	52
% of corals out of the breeding population in which sex change occurred	66 (n = 64)	11 (n = 6)	19 (n = 10)
% of corals out of the breeding population in which repetitive sex change occurred	29 (n = 28)	0	10 (n = 5)
Avg. W(g) at sex change	1067 g ± 141	884 g ± 395	605 g ± 285
Range of W(g) at sex change	195–2400 g	330–1390 g	232–968 g
Avg. W(g) of an individual in the population	779 g ± 53	630 g ± 59	437 g ± 25
W(g) range of the population	25–2450 g	22–2580 g	32–2039 g
W(g) of largest reproductive coral	2400 g	2580 g	1866 g
W(g) of the largest coral	2450 g	2580 g	2039 g
W(g) at first reproduction	40 g	41 g	32 g

Summary of the main results obtained during 2004–2017 at Okinawa, Aqaba, and Eilat. Asterix (*) indicates populations whose reproduction had been studied for two years or more. Bidirectional – where sex change occurred in both directions, either from female to male or from male to female, repetitive sex change - when repeated events of sex change occurred in the same individual.

### Size frequency distribution and sex ratio

**Figure 3:**
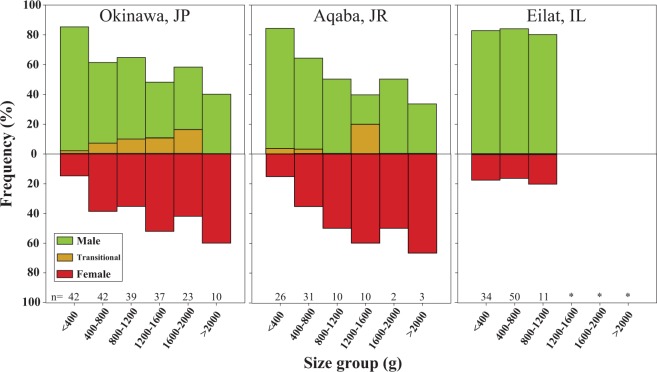
*Herpolitha limax*: Size-frequency distribution of individuals for which sex was determined. Since individual corals in the population changed sex frequently, the calculation of size-frequency distribution was performed as an accumulation of data from all years. Data represent results from 2012 onward: Okinawa, Japan (2014–2017), Aqaba, Jordan (2013, 2015) and Eilat, Israel (2012–2017). Number of individuals (n) within each size group is indicated under the bars. Size groups (>1200 gr.) in Eilat contained only one individual indicated by (*) and are not shown in the figure.

The size (weight) of individuals at first reproduction was similar at all sites and varied from 32 g in Eilat to 41 g in Okinawa (Table 2). However, In Eilat and Aqaba females were lighter in weight (weighting 169 g and 133 g, respectively) than in Okinawa, where the smallest female measured weighed 380 g. Nonetheless, the smallest weight group (<400 g) at all sites was strongly biased towards males (χ^2^ test, χ^2^ = 46.835, p < 0.001); the frequency of males in the small-size groups was higher than that of females (Fig. 3). Hence, the primary sex (i.e. smallest reproducing individuals) is male indicating that the direction of sex change in this species is that of protandry. In Okinawa, the weight (g) of corals documented as females during the study, including the sex-changing individuals, was significantly higher than that of consistent males (Kruskal-Wallis test, Q = 3.051, P < 0.05) similar to the trend observed in Aqaba (Mann-Whitney test, T = 689.5, P = 0.013). The weight of corals in the ‘transitional state’ was also significantly heavier than that of consistent males (Kruskal-Wallis test, Q = 2.744, P < 0.005). In contrast, at Eilat no significant difference was found between the weight of females, which include the sex-changing corals, and that of consistent males (Mann-Whitney test, T = 361, P = 0.845) (Fig. 2 boxplots, Supplementary Material Figs S3, S3; boxplots).

**Figure 4:**
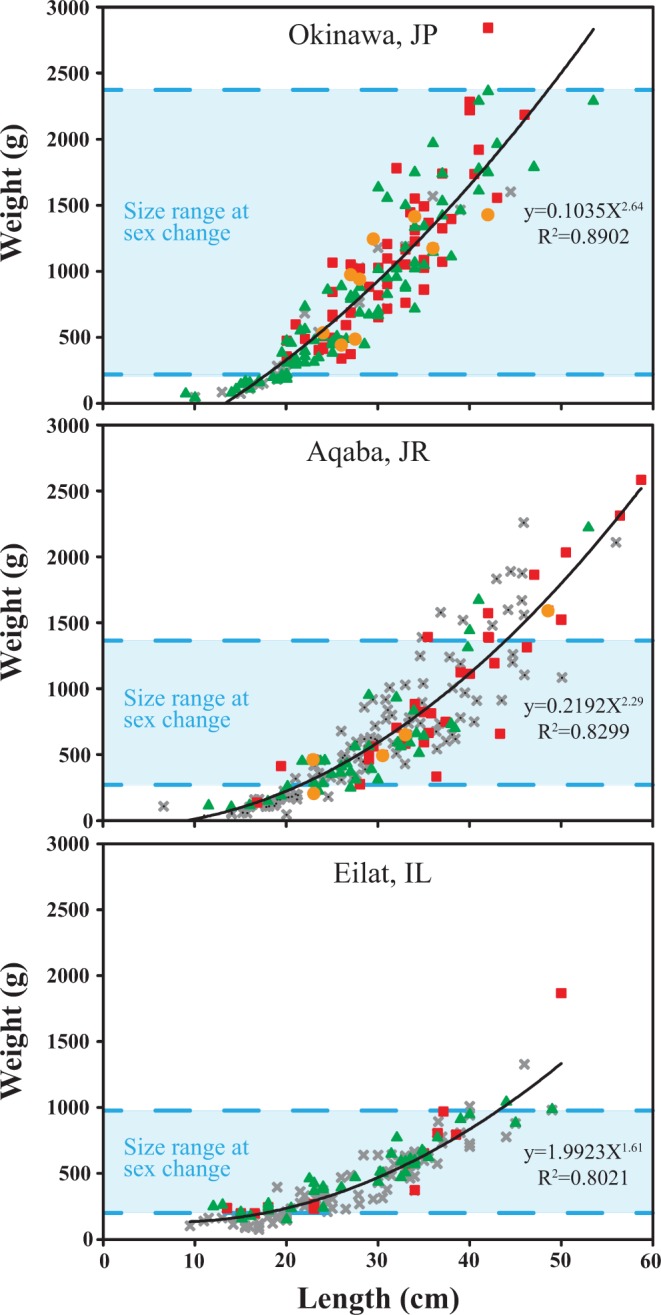
Allometric relationship and size range at sex change of the monitored *H. limax* populations at Okinawa, Japan (2004–2017); Aqaba Jordan (2013–2016); and Eilat, Israel (2009–2017). Green triangles, males (M); red squares, females (F); gray X, non-reproductive (NR); orange circles, transitional stage (T). Number of individuals n (Okinawa) = 180, including 93 M, 57 F, 21NR and 9 T; N (Aqaba) = 178, including 39 M, 31 F, 103NR and 5 T; N (Eilat) = 157, including 45 M, 15 F and 97NR.

The average size of the population and the weight range of individuals at which sex change occurred differed among the study sites (Fig. 4, Table 2). The average weight of the *H. limax* population in Eilat was the smallest, 437 g ± 25 (mean ± 95% confidence interval), and sex change in Eilat also occurred at a relatively small average weight (605 g ± 285) and within a narrow range (232–968 g). In Aqaba, the average weight of the population was 630 g ± 59, and sex change occurred at an average of 885 g ± 395 and ranged between 330 to 1,390 g. In Okinawa, the population was significantly heavier than that of both Eilat (Kruskal-Wallis test, Q = 9.030, P < 0.05) and Aqaba (Kruskal-Wallis test, Q = 4.224, P < 0.05) with an average weight of 779 g ± 53. Additionally, sex change occurred at a larger average weight (1,067 g ± 141) and within a much wider range 195–2400 g. The largest reproductive coral in Eilat was a female weighing 1,866 g; in Aqaba also a female, weighing 2,580 g; and in Okinawa a female and a male, both weighing 2,400 g (Table 2).

Sex-ratio of *H. limax* populations varied significantly between Aqaba and Eilat (Pairwise χ^2^ test, p < 0.001) and between Okinawa and Eilat (Pairwise χ^2^ test, p < 0.001). However, no significant difference was found between Okinawa and Aqaba (Pairwise χ^2^ test, p = 0.648). In Eilat, sex-ratios were male-biased, varying from 1:0 (M:F) in 2012, which represents the most male-biased year, to 1:0.6 (M:F) in 2013, which represents the least male-biased year, reflecting significant differences between different years (χ^2^ test, χ^2^ = 17.27, p < 0.01). In Aqaba and Okinawa sex ratios included individuals that released both male and female gametes within the same reproductive season (i.e. transitional state, T) and were relatively less biased towards maleness (Fig. 2, Supplementary Material Fig. S1). In Aqaba, the sex ratio was 1:0.8:0.09 (M:F:T) in 2013 and 1:0.5:0.11 (M:F:T) in 2015; and in Okinawa, it was 1:0.2:0 (M:F:T) in 2014, 1:0.65:0.08 (M:F:T) in 2015, 1:0.62:0.28 (M:F:T) in 2016, and 0.88:1:0.11 (M:F:T) in 2017, which represents the only year in which sex ratio was female biased (Table 1). No significant differences were found among years in Aqaba (χ^2^ test, χ^2^ = 0.963, p = 0.353). However, in Okinawa, sex ratio did vary significantly among years (χ^2^ test, χ^2^ = 24.387, p < 0.01).

### Average annual increase in growth

**Figure 5:**
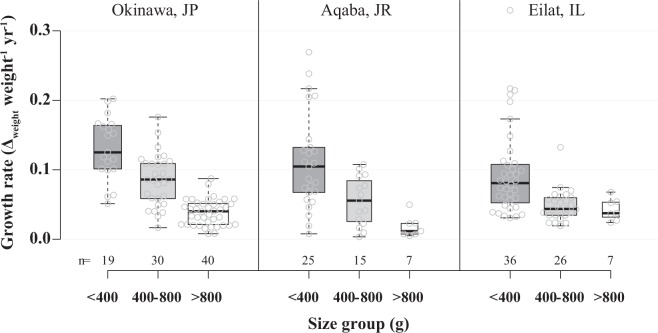
*Herpolitha limax*: average annual percentage increase in growth rate (weight) in different size groups, as measured at Okinawa, Japan (2004–2017); Aqaba, Jordan (2013–2016); and Eilat, Israel (2009–2017). Boxplot centerlines show the medians; box limits indicate the 25^th^ and 75^th^ percentiles; whiskers extend 1.5 times the interquartile range from the 25^th^ and 75^th^ percentiles; data points are represented by blank gray circles. Number of individuals (n) within each size group is indicated under the box.

The average annual percentage increase in growth (weight) was calculated following equation 1 for the different size groups. The smallest group’s (<400 g) growth rate was significantly higher than that of the intermediate group (Two-way ANOVA, q = 9.322, P < 0.001), as well as the largest group (Two-way ANOVA, q = 11.610, P < 0.001) (Fig. 5). Among the study sites, the *H. limax* population in Okinawa demonstrated the highest growth rate, being significantly higher than at both the Eilat (Two-way ANOVA, q = 3.912, P = 0.016) and Aqaba (Two-way ANOVA, q = 3.762, P = 0.021) study sites.

## Discussion

Theories related to sex change have been attempting to predict the conditions favoring sex change, the type of change, and its timing. The repetitive sex change displayed by the coral *Herpolitha limax*, distributed in widely divergent geographical areas and exhibiting substantial within habitat spatial distributions, enabled examination of the interplay between environmental conditions and sex allocation, and its manifestation in a sex-changing coral species.

### Repetitive sex change

While the vast majority of scleractinian corals are simultaneous hermaphrodites^20,21,34,35^, all corals whose reproduction has been studied within the family Fungiidae have been reported to be gonochoristic^13,20,23,34,36–38^. Of these, at least four fungiid species were found to be protandrous sequential hermaphrodites; that is, the polyps function as solely male when small and as solely female when larger^13,38^ in accord with the sex-allocation theory^3^. Two of these species (*C. echinata* and *C. crassa*) can change sex bi-directionally^13^ (i.e. either from female to male or from male to female in the same population). Here we report, for the first time in corals, on a repetitive (multiple) pattern of sex change exhibited by the fungiid coral *H. limax*. We found *H. limax* to be a protandrous species with the ability to execute multiple events of sex change and presenting reproductive tactics analogous to those of plants, which exhibit labile sexuality in response to energetic and/or environmental constraints. Individuals of *H. limax* released male gametes during their early life stages (i.e. protandrous), probably due to the overall low energetic state of small corals (i.e. lower capacity to obtain enough energy to support daily energetic requirements; e.g., maintenance, growth and higher energetic costs of being a female), and released male and female gametes alternately at intermediate and large sizes. A low percentage of corals released both male and female gametes within the same reproductive season in Aqaba and in Okinawa (Table 1). We adopt the definition that sex change encompasses organisms that simultaneously perform both sex functions during the transitional stage while changing from one sex to the other^5^. We term this exceptional reproductive state as a ‘transitional state’ (T). This reproductive state featured more frequently in the intermediate individual sizes (Fig. 3), the main size group that demonstrated sex change.

The evolution of one-way sex change has been successfully explained by the size-advantage model, which predicts that an individual should change sex when the reproductive value (i.e. a measure of the expected future reproductive success) of the other sex exceeds that of its present sex^31^. Following the same theoretical concept, if the reproductive value between the sexes changes more than once during a lifetime, bi-directional sex change (i.e. the occurrence of sex change either from female to male or from male to female) will be favored. Such an energetically costly exchange can occur only if a reduction in body size or in resource availability for reproduction takes place in the course of the organism’s lifetime^39^. Causes for such reduction could be fluctuations in local environmental conditions. For example, more females than males of an epiphytic orchid occur under open canopies in comparison to shady areas^40^. Similarly, in plants, males were shown to be proportionately more abundant during periods of poor environmental conditions, in comparison to periods of improved conditions^16–18^. Alternatively, a reduction in reproductive value could result from the cost of reproduction itself. Female jack-in-the-pulpit plant, which were male when small, lost weight following reproduction and sometimes became smaller than the critical size advantageous to reproduce as females, consequently changing back to males^41^. A similar pattern was documented in the polychaete *Ophryotrocha puerilis* (Dorvilleidae). In this species, the larger individual in a pair adopts the female role until outgrown by the faster-growing male, leading to repeated episodes of sex change by both pair members^5,42^. Following similar energy budget considerations, Loya and Sakai^13^ described sex change in intermediate sizes of the coral *C. echinata*, and suggested that corals could ‘recover’ from the relatively costly female reproduction by channeling energy to the less costly male reproduction in alternate years, and thereby increasing their reproductive success. In addition to the internal codes (i.e. genetic, developmental) inherent within the individual, environmental factors may act as a catalyst towards the evolution of specific adaptive traits of sexual reproduction. Consequently, we suggest that the multiple occurrences of sex change exhibited by *H. limax* constitute a flexible response of individual specimens to local environmental conditions enabling them to increase their reproductive success (fitness). Expressing female sexuality may follow improved environmental conditions and increased energy resources; while expressing the opposite sex, in alternate years, may compensate for an overall low energetic state caused by stress and/or for the high energetic cost of the preceding female reproductive state.

### Effects of the environment on resource allocation to reproduction, growth, and sex expression

Human-induced perturbations have left a strong ecological imprint on the coral reefs at all the study sites^43–46^. However, the basic ecological features as well as the intensity and prevalence of anthropogenic and natural perturbations differ among the sites (see Environmental differences in the Methods) with the coral reef of Okinawa seemingly providing the most favorable conditions for fungiid species. In spite of the massive bleaching events that resulted in a reduction of coral cover and species richness^47–52^, as well as other man-made and natural disturbances, such as freshwater runoff and typhoons^46^, the fungiid corals in Okinawa demonstrate high reproduction rate, appear in a good condition and are very abundant^13,23^. In contrast, the fungiid corals in the GoA/E are not common^53^, attain smaller size (Table 2), and were documented to suffer from diseases (L.E-S., YL, pers. obs.). Nonetheless, the repetitive sex change displayed by the coral *H. limax* is shared across all three spatially distant study sites. Furthermore, both seasonality and timing of spawning are synchronized among the sites (i.e. 8–10 am, for 5–7 consecutive days, five days after the full moon of June-August or July-September, depending on the lunar periodicity of the given year).

The differences among sites in the mechanisms that can potentially affect the species’ life-history traits (i.e. population density, environmental conditions) are reflected in several characteristics of the *H. limax* populations. Thus, the sporadically scattered populations in Eilat and Aqaba exhibited significantly slower growth (One-way ANOVA, F = 5.552, P = 0.005), earlier sex change, and lower percentages of reproduction and sex change in comparison to the densely aggregated populations in Okinawa. At all sites, sex ratio varied among years, but almost always was biased towards maleness and growth rate decreased with size. (Fig. 5). In clonal organisms such as scleractinian corals, growth and size may be genetically indeterminate^54^ and consequently, may more reflect the local environmental conditions rather than being merely an innate characteristic of the species. We posit that some variations in such life-history traits may largely stem from local environmental conditions, which have a direct impact on their energetic constraints. The higher overall energetic state of the corals in Okinawa is evident also in their higher percentages of reproduction and sex change (both being energetically costly functions) in comparison with their GoE/A conspecifics (Table 1). Similar to the *Eurya japonica* shrub, which changed sex more frequently with higher growth rates and more exposure to light throughout the year^19^, the higher percentages of reproduction and sex change documented in the *H. limax* population in Okinawa are correlated with higher growth rates (i.e. in comparison with the Eilat and Aqaba populations) (Tables 1, 2). In accordance with sex allocation theory, which predicts a bias in sex ratio toward the first sex (i.e. the first sex expressed by an individual during its life cycle)^3,27^, the sex ratio exhibited by the coral *H. limax* at all the study sites, (with one exceptional year in Okinawa), was always biased towards maleness. However, large fluctuations were documented among sites and within the same site during consecutive years. The difference in the *H. limax* sex ratio between Aqaba and Eilat, which are only 4.5 km apart, is particularly striking (Table 1). Hence, we suggest that in the case of *H. limax*, as in many dioecious plants^18^, sex ratio may be a consequence of a synergistic flexible response by individuals to local environmental conditions and to population demography, since sex ratios often differ for stacked (Okinawa) versus widely dispersed (Eilat and Aqaba) coral populations (see Collin and Promislow^12^). At the same time, we argue that the relatively lower abundance of females in Eilat reflects the population’s weakened physiological state, since maintaining female reproduction is considered more energetically costly than male reproduction^13^.

An early investment predominantly in sperm production allows reproduction to commence without the initial expense of egg production, perhaps so that colony growth can continue to a larger, safer size^54^. However, species that aim to overcome environmental stress will probably invest more in reproduction than in any other energy-costly life function^55^. The energy allocation towards reproduction may be enabled through the inducement of reproductive investment early on in life^2^. Even though first reproduction was documented at similar sizes at all the studied sites, the primary sex stage (i.e. solely male) in Eilat and Aqaba was shorter than in Okinawa (Table 2, Fig. 4). One possible explanation is that in order to maximize their reproductive potential, the *H. limax* population in the GoA/E allocate energy also to female reproduction at a relatively early age, before the accumulated risk of mortality becomes excessively high. This assumption takes into consideration also the lower growth rate of the corals in the GoE/A (Fig. 5), as well as the average smaller size of individuals in that population (Table 2). Hence, indicating a lower energy allocation to that specific life function (i.e. growth) probably in favor of other life functions, and presumably a shorter life span of an individual coral. However, the specific niche exploited by a species and its local spatial distribution characteristics, which differ between *H. limax* populations in Okinawa and the GoE/A, may also produce such a pattern. In very low-density populations, reproduction may be hampered by a shortage of potential mates^56^. This may have led the corals in the GoE/A to enhance female reproduction earlier than would have been expected in a more benign environment, such as in Okinawa. However, to confirm this assumption, a rigorous examination of small-sized individuals, subjected to various conditions, is necessary.

Trade-offs between alternative life-history traits depend on the available resource pool and may be expressed at higher taxonomic level (population) as well as at the level of individuals within a population^7^. The differences in the life history characteristics of *H. limax* were found to be more substantial between the geographically distant sites, but were also evident between the two close sites of the GoE/A, the Aqaba reefs are relatively less perturbed than those of Eilat^43,44,57^. Nevertheless, to confirm the possible coupling between the proximate mechanisms that determine patterns of sex change and environmental factors, additional species-specific studies of sex-changing organisms are required.

### Timing of sex change

In an attempt to understand the variation in timing of sex change, Warner^31^ suggested that the Size Advantage Hypothesis (SAH) be framed in terms of ‘reproductive value’, which is a measure of the expected future reproductive success, taking into account effects of growth rate and mortality, and is often dependent on the local environment, local demography, and the individual’s own status. Theory predicts that sex change will occur earlier in populations exhibiting slower growth rates and higher mortality rates^3,7,10,24–26,58^. Indeed, sex change did occur earlier and in a narrower size range at the Eilat and Aqaba study sites, where growth rate was significantly lower (Fig. 5; Table 2). Because the expected reproductive success depends on both size and/or age and the pool of each animal’s potential mates, the optimal size at sex change should vary in response to the size distribution or age structure of the population^12^. Accordingly, the time and range of sex change in the GoE/A may also be attributed to the scattered spatial distribution pattern of the species, and is compatible with the finding that densely stacked *H. limax* individuals (Okinawa) change sex at larger sizes than their spatially distant conspecifics (Eilat and Aqaba) as previously suggested by Collin and Promislow^12^. When reporting on bidirectional sex change in the fungiid species *F. repanda* and *C. echinata*, which inhabit the same patch reef in Okinawa and hence are subjected to the same environmental conditions, Loya and Sakai^13^ found variance in the timing of sex change between the two species. That in *F. repanda* occurred at a relatively small average weight and within a narrow range, compared to *C. echinata*, and the average increase in growth was significantly lower in *F. repanda*. Similarly, the size (weight) of sex change in the Aqaba population varied from that of the Eilat population: corals changed sex at a relatively smaller size in Eilat (605 g ± 285) than in Aqaba (885 g ± 395), and growth rate was slower in Eilat (Table 2). Differences in the relative frequency of sex change can even occur on neighboring reefs, indicating that individuals select a life-history tactic that may or may not involve adult sex change, depending on the mating conditions that they experience in that habitat patch^7^. We posit that in fungiid corals the timing of sex change may be determined by the combination of a chemical response to nearest-neighbor conspecifics cues (e.g. pheromones release in stacked coral populations, such as in Okinawa), as well as by local environmental conditions.

The findings of this study contribute to a better understanding of the trade-offs among life-history traits in modular organisms, and suggest that the repetitive sex change displayed by the fungiid coral *H. limax* is environmentally mediated and phenotypically plastic, analogous to that in sexually labile plants. Much of the data obtained in this work provide support for the sex allocation theory^3^. Although uncommon, these exceptional reproductive tactics must be considered if we are to fully understand the evolution of sexual systems in corals. We further argue that fungiid corals provide an excellent model organism by which to study the mechanism of sex change in animals, due to their unique life form, which enables a relatively easy and accurate determination of various life-history parameters.

## Methods

### Study sites

This study was variously conducted over a period of 3–13 years and took place at three geographically distant sites: Okinawa (Japan) at several patch reefs near Sesoko Marine Station, (26°39′N, 127°52′E; during 2004–2017); Eilat (Israel) on the western side of the Gulf of Eilat/Aqaba (29°30′N, 034°55′E; during 2009–2017); and Aqaba (Jordan), on the eastern side of the GoE/A (29°27′N, 034°58′E; during 2013–2016).

The island of Okinawa is part of the Ryukyu Archipelago in Japan, which defines the boundary between the East China Sea (west) and the Philippine Sea (east). The archipelago hosts a diverse coral community, with species numbers comparable to those found in the Great Barrier Reef ^59,60^. In spite of the massive bleaching events that resulted in a reduction of coral cover and species richness^47–52^, as well as other man-made and natural disturbances such as polluted freshwater runoff and typhoons^46^, the reefs in Okinawa are continuous and massive with a relatively high coral cover^57^. This may be a consequence of the influence of the Kuroshio current, which introduces warm tropical waters from the Philippines and equatorial Pacific into the archipelago^60^. The Gulf of Eilat/Aqaba (GoE/A; 180 km by 20 km) is a semi-enclosed sea, and is connected to the open ocean via a narrow, shallow (137 m) strait at its southernmost end (Bab el Mandeb)^61^.

The coral reefs of the GoE/A are situated at the northern limits of global coral-reef distribution and embrace a rich diversity of habitats that include shallow coastal lagoons, sea grass beds, mangrove stands, and the coral reefs themselves^43^. Due to the steep bathymetry of the area, coral-reef communities are limited to a narrow, patchy band along most of the coast^45,62^. Throughout the last few decades, the cities of Aqaba and Eilat, situated at the tip of the bay, have been experiencing an enormous growth, with increased coastal development^43,45^ that has resulted in substantial stress on the coral-reef ecosystems in the GoE/A. However, in spite of the close proximity of the Aqaba reefs to the Eilat reefs (4–5 km), the extent of deterioration varies between these sites. While the reef of Eilat is considered highly degraded with extreme deterioration in coral abundance and living cover^43,44,63,64^, the coral reefs in Jordan are described as being in good condition with high coral diversity and a relatively low rate of coral mortality^45,65^. These differences have also been attributed to the flow dynamics. The area is interconnected by surface currents that generally flow in a clockwise direction from Saudi Arabia up the Jordanian coast and then down the Israeli coast^66,67^. According to Labiosa *et al*.^68^, Ekman-driven down-welling along the western side (Eilat) can potentially lead to more oligotrophic conditions, while the upwelling along the eastern margin (Aqaba) generates mesotrophic to eutrophic conditions for much of the year. These dynamics coincide with higher than average Chlorophyll *a* concentration and cooler surface seawater temperatures on the eastern side of the GoE/A.

The study sites are characterized by different environmental conditions and by divergent densities of the *H. limax* populations. In both the Eilat and Aqaba reefs *H. limax* populations appear in a dispersed manner and occur mostly at a depth range of 5–50 m, albeit at a higher frequency in Aqaba than in Eilat (L.E-S., YL, pers. obs). In contrast, in the coral reefs of Okinawa, the *H. limax* population is mainly concentrated at few sites that are inhabited by tens of thousands of specimens (probably more than 12 fungiid species), densely distributed between 3 to 20 m depth^13^. Surface seawater temperatures in Okinawa and in the GoE/A are quite similar; varying from 20 to 28 C° in Okinawa^48^ and from 21 to 28 C° in the GoE/A^53^. However, the corals in Okinawa were subjected to recurring bleaching events in the past few decades^48^, whereas in the GoA/E no bleaching events were documented and corals are considered ‘resistant’ to high temperatures^61^.

### Specimen collection

At all three sites, specimens of *H. limax* of all possible size groups were collected from the reef and transferred to an outdoor seawater system at the local laboratory. Each sampled coral was tagged individually with a numeric plastic tag attached by a nylon fishing cord inserted through a thin hole drilled at the edge of the coral skeleton using a portable drill^13^. No adverse effects on the corals were observed following the tagging procedure. All corals were measured (length and weight) and photographed immediately after collection. A ruler was placed next to each photographed specimen to enable scale (Fig. 1a). The corals were kept in large containers with constant seawater inflow and were transferred to separate individual aquaria for spawning observations, in accordance with the expected timing of reproduction (see Supplementary Material for further information on the discovery of spawning time). Following each annual set of measurements and observations, the tagged corals were returned to their natural habitat and retrieved the following year, one to two weeks before the expected breeding season. Sample sizes of the different coral size groups were increased over the years in order to enable a more in-depth examination of the data accumulating from the observations.

### Reproduction studies

Observations on *H. limax* started in 2004 at the Sesoko Marine Station, Japan, as part of a wider research project of studying reproductive strategies in the Fungiidae^13^. During the initial stages, 27 *H. limax* individuals, representing different size groups, were collected from the reef and placed in separate aquaria to spawn. Nightly observations were made between 5 pm and a few hours before dawn, for eight consecutive nights after the full moon in July 2004. However, despite revealing the reproduction of ten other fungiid species^13,23^, no reproduction was recorded for *H. limax*. Over the years, sample size was increased and observation times were changed in an attempt to determine the exact reproductive season and spawning time of the same individually tagged individuals. The observational procedure was repeated during August 2006, June to September 2007, 2010, 2014–2017 in Okinawa; June to September 2009–2017 in Eilat; and June to September 2013–2016 in Aqaba. Coral sex was determined from the unique shape of the shed gametes and was verified using a microscope.

### Growth rate

Growth rates of individuals were monitored by means of annual size measurements of individual corals. Using a tape measure, length (L, mm) was measured to the nearest 1 mm; weight (W, g) was measured to the nearest 1 g, following careful removal of excess moisture (Fig. 1a). Growth rate was studied in all three sites: Okinawa, Japan (2004–2017); Aqaba, Jordan (2013–2016); and Eilat, Israel (2009–2017), and calculated according to equation 1:

1 $$\begin{array}{c}{\rm{Individual}}\,{\rm{annual}}\,{\rm{growth}}\,{\rm{rate}}\,({\rm{increas}}\,{\rm{in}}\,{\rm{weight}}\,({\rm{g}}))=\frac{{\rm{\Delta }}{\boldsymbol{Wx}}({\bf{g}})/{\boldsymbol{N}}}{{\boldsymbol{Wx}}}\\ {W}{\rm{x}}-{\rm{Weight}}\,({\rm{g}})\,{\rm{of}}\,{\rm{individual}}\,{\rm{coral}}\,{\rm{x}}\\ N\,\mbox{--}\,{\rm{Total}}\,{\rm{number}}\,{\rm{of}}\,{\rm{years}}\,{\rm{between}}\,{\rm{consecutive}}\,{\rm{weight}}\,{\rm{measurements}}\\ {\rm{\Delta }}Wx\,(g)-{\rm{Weight}}\,{\rm{difference}}\,({\rm{g}})\,{\rm{between}}\,{\rm{the}}\,{\rm{initial}}\,Wx\,{\rm{and}}\,{\rm{its}}\,{\rm{weight}}\,{\rm{after}}\,{\rm{N}}\,{\rm{years}}.\end{array}$$

### Statistical analyses

Statistical analyses were performed using R software^69^ and Sigmaplot 12.2 (Systat Software, Inc). Data were checked for normality (Shapiro-Wilk normality test) and homogeneity of variance (F-test) and tested accordingly with appropriate tests. Post-hoc tests were carried out by Tukey and Holm tests. P-values < 0.05 were considered statistically significant. Heat-maps were created in R using *Superheat* package^70^.

## Supplementary information

Supplamentary info 1

## Data Availability

The data that support the findings of this study are available from the corresponding authors on request.
