# Supplementary material for: Repetitive sex change in the stony coral Herpolitha limax across a wide geographic range
Source: Sci Rep. 2019 Feb 27;9:2936. doi: 10.1038/s41598-018-37619-y (PMC6393666; doi:10.1038/s41598-018-37619-y)
Supplement: Supplementary file 1 — Supplamentary info 1 [file 41598_2018_37619_MOESM1_ESM.pdf]

Repetitive sex change in the stony coral *Herpolitha limax* across a wide geographic range

Lee Eyal-Shaham[1,2,*], Gal Eyal[1,2,*], Kazuhiko Sakai[3], Yoko Nozawa[4], Saki Harii[3], Frederic Sinniger[3], Omri Bronstein[1], Or Ben-Zvi[1], Tom Shlesinger[1] and Yossi Loya[1,*]

[1] Tel Aviv University, School of Zoology, Tel Aviv 6997801, Israel

[2] The Interuniversity Institute for Marine Sciences, P.O. Box 469, Eilat 8810369, Israel

[3] Tropical Biosphere Research Center, University of the Ryukyus 3422 Sesoko, Motobu, Okinawa 905-0227, Japan

[4] Biodiversity Research Center, Academia Sinica, Taipei, Taiwan

Supplementary Information

***Herpolitha limax* – discovery of spawning time**

In accordance with what was then (2004-2010) known on the time of reproduction of fungiid corals, observations took place every two hours, for seven consecutive nights starting five days after the full moon of June, July and August, from 5 pm until two hours before dawn (see detailed methodology in [1,2]). Nevertheless, throughout six years of repeated observations on the same *H. limax* individuals no spawning was recorded. During those years, ten other fungiid species that were monitored annually spawned occasionally throughout the night [2]. In August 3, 2010, after accidentally having left the monitored corals in their individual aquaria until the following morning, we recorded spawning for the first time in three males and two females. However, our unfortunately incorrect interpretation, at the time, resulted in following the dogma of night spawning; and, therefore, we did not change our observation protocol. Only in July 2012, in Eilat, Israel, after gradually extending the observation hours to 10 am, did we actually witness, for the first time, by chance, daytime spawning (09:00) and consequently confirmed *H. limax* as a day-spawner.

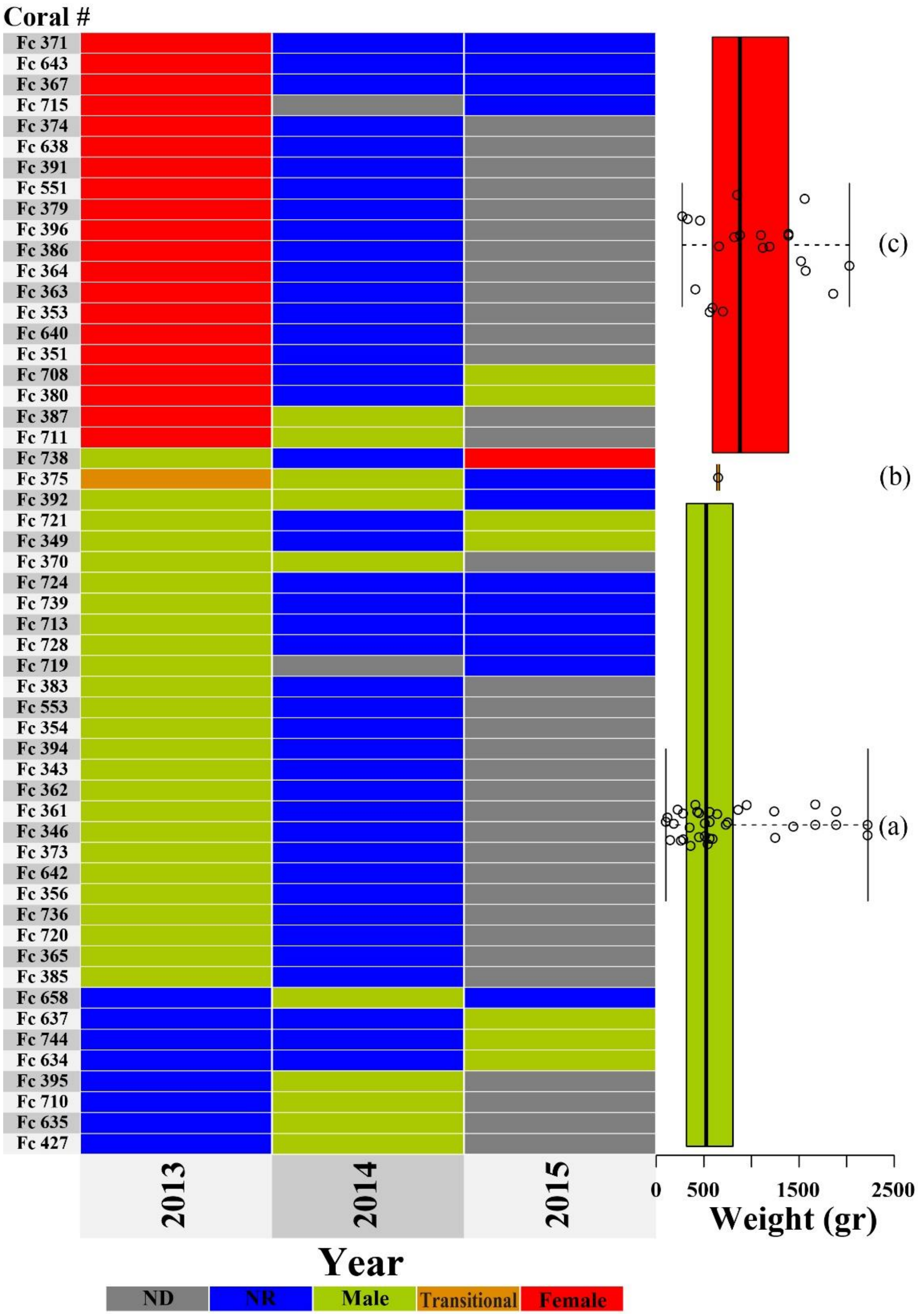

Figure S1: *Herpolitha limax* (Aqaba, Jordan): A heat-map displaying 'expressed sexuality' per individual coral (i.e. corals whose reproduction had been studied for two years or more) throughout the study years. The cluster-aggregated boxplots to the right of the heat-map display the individual coral weight (g). Centerlines show the medians; box limits indicate the 25th and 75th percentiles; whiskers extend to min and max values and the range is colored in accordance with the legend below the heat-map; data points are represented by blank circles. N (from top to bottom) = 21, 1, 32 individual coral weights. The color of each cell in the heat-map corresponds to the legend below it. Gray cells (ND) correspond to missing data and blue cells (NR) to non-reproductive coral. The rows (individual corals) are ordered by patterns of sexuality. Boxplot a – represents individuals that were documented only as 'males' throughout the study; Boxplot b –

represents the single individual in Aqaba that was documented to contain both male and female gametes simultaneously; Boxplot c - represents individuals that were documented as 'females' throughout the study. Due to the small number of individuals that changed sex, we included these individuals in the female group.

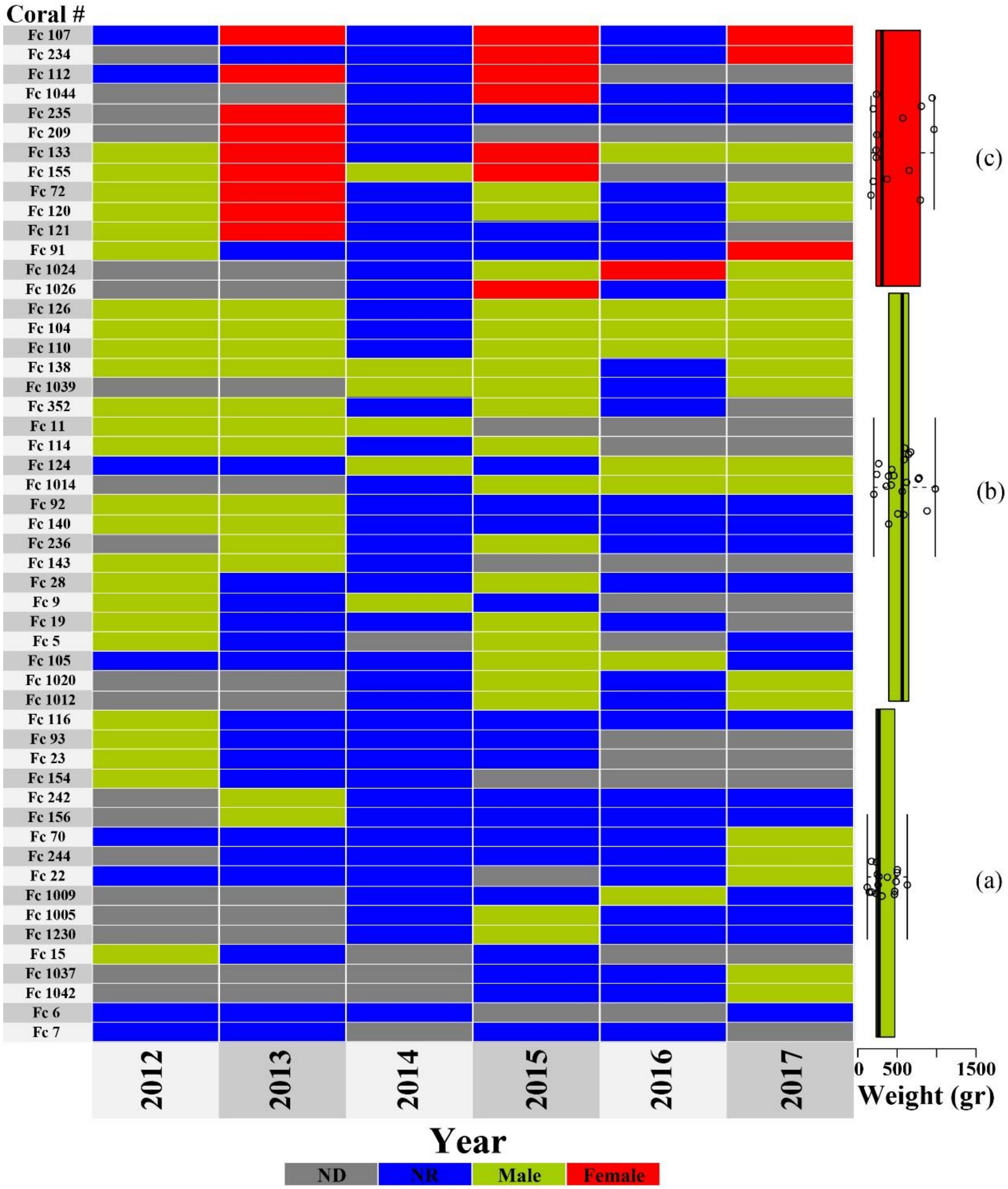

Figure S2: *Herpolitha limax* (Eilat, Israel): a heat-map displaying ‘expressed sexuality’ per individual coral (i.e corals whose reproduction had been studied for two years or more) throughout the study years. The cluster-aggregated boxplots to the right of the heat-map display the individual coral weight (g). Centerlines show the medians; box limits indicate the 25th and 75th percentiles; whiskers extend to min and max values and the range is colored in accordance with the legend below the heat-map; data points are represented by blank circles. n = (from top to bottom) 14, 21, 17 individual coral weights. The color of each cell in the heat-map corresponds to the legend below it. Gray cells (ND) correspond to missing data and blue cells (NR) to non-reproductive coral. The rows (individual corals) are ordered by patterns of sexuality. Boxplot a -

represents individuals that were documented as 'males' only once throughout the study; Boxplot b - represents individuals that were documented as 'males' more than once throughout the study; Boxplot c - represents individuals that were documented as 'females' during the study. Due to the small number of individuals that changed sex, we included these individuals in the female group

References

1 Loya, Y. & Sakai, K. Bidirectional sex change in mushroom stony corals. *Proceedings of the Royal Society of London B: Biological Sciences* **275**, 2335-2343 (2008).

2 Loya, Y., Sakai, K. & Heyward, A. Reproductive patterns of fungiid corals in Okinawa, Japan. *Galaxea, Journal of Coral Reef Studies* **11**, 119-129 (2009).
